# Machine learning prediction of hematoma expansion in acute intracerebral hemorrhage

**DOI:** 10.1038/s41598-022-15400-6

**Published:** 2022-07-21

**Authors:** Satoru Tanioka, Tetsushi Yago, Katsuhiro Tanaka, Fujimaro Ishida, Tomoyuki Kishimoto, Kazuhiko Tsuda, Munenari Ikezawa, Tomohiro Araki, Yoichi Miura, Hidenori Suzuki

**Affiliations:** 1 Department of Neurosurgery, Mie Chuo Medical Center, 2158-5 Myojin-cho, Hisai, Tsu, Mie 514-1101 Japan; 2 Department of Neurosurgery, Matsusaka Chuo General Hospital, 102 Kobo, Matsusaka, Mie 515-8566 Japan; 3 Department of Neurosurgery, Suzuka Kaisei Hospital, 112-1 Ko-cho, Suzuka, Mie 513-8505 Japan; 4 Department of Neurosurgery, Mie University Graduate School of Medicine, 2-174 Edobashi, Tsu, Mie 514-8507 Japan

**Keywords:** Neuroscience, Neurology

## Abstract

To examine whether machine learning (ML) approach can be used to predict hematoma expansion in acute intracerebral hemorrhage (ICH) with accuracy and widespread applicability, we applied ML algorithms to multicenter clinical data and CT findings on admission. Patients with acute ICH from three hospitals (n = 351) and those from another hospital (n = 71) were retrospectively assigned to the development and validation cohorts, respectively. To develop ML predictive models, the k-nearest neighbors (k-NN) algorithm, logistic regression, support vector machines (SVMs), random forests, and XGBoost were applied to the patient data in the development cohort. The models were evaluated for their performance on the patient data in the validation cohort, which was compared with previous scoring methods, the BAT, BRAIN, and 9-point scores. The k-NN algorithm achieved the highest area under the receiver operating characteristic curve (AUC) of 0.790 among all ML models, and the sensitivity, specificity, and accuracy were 0.846, 0.733, and 0.775, respectively. The BRAIN score achieved the highest AUC of 0.676 among all previous scoring methods, which was lower than the k-NN algorithm (p = 0.016). We developed and validated ML predictive models of hematoma expansion in acute ICH. The models demonstrated good predictive ability, showing better performance than the previous scoring methods.

## Introduction

Hematoma expansion occurs in one third of patients with acute intracerebral hemorrhage (ICH), and has been identified as a factor associated with early neurologic deterioration and poor outcome^1–5^. Therefore, its accurate prediction on admission assists in developing appropriate patient management strategies. Various predictive factors for hematoma expansion have been suggested, including time from onset to baseline imaging, older age, antiplatelet use, anticoagulant use, ICH volume on baseline imaging, and CT markers such as intrahematoma hypodensities, irregular hematoma shape, blend sign, and CT angiography spot sign^3–15^. Additionally, several predictive scores that combine those factors have been reported^16–20^.

Machine learning (ML) approaches have been used in clinical studies, and perform well in disease detection, outcome prediction, and classification of various medical data^21–24^. To apply the study results using ML to clinical practice, there are some important points to be considered. The Radiological Society of North America developed a list of key considerations of ML research: it emphasized the generalizability of the research work and the reproducibility of the work’s results^25,26^. However, many clinical studies using ML lack those perspectives: for example, single-vendor images are used in imaging analysis, and ML algorithms are not publicly available^25,26^.

To develop accurate, generalizable and widely applicable predictive models of hematoma expansion in acute ICH, we applied ML algorithms to clinical data and CT findings on admission. Multicenter data and multivendor CT images were used, and the algorithms were made available via a website.

## Materials and methods

### Study population

Consecutive patients with acute ICH who were admitted to Mie Chuo Medial Center between December 2012 and July 2020, Matsusaka Chuo General Hospital between January 2018 and December 2019, Suzuka Kaisei Hospital between October 2017 and October 2019, and Mie University Hospital between January 2017 and July 2020 were retrospectively reviewed. Patients in Mie Chuo Medical Center, Matsusaka Chuo General Hospital, and Suzuka Kaisei Hospital were assigned to the development cohort, and those in Mie University Hospital were assigned to the validation cohort.

Inclusion criteria were defined as follows: ≥ 18 years of age; baseline CT scan within 24 h of onset; and follow-up CT scan within 30 h after baseline CT scan. Exclusion criteria were defined as follows: traumatic ICH; secondary cause of ICH (e.g., aneurysm, arteriovenous malformation, arteriovenous fistula, hemorrhagic transformation of infarction, and tumor); and surgical evacuation before follow-up CT scan.

Baseline clinical variables included age, sex, medical history (ICH, cerebral infarction, ischemic heart disease, hypertension, diabetes mellitus, and dyslipidemia), anticoagulant use, antiplatelet use, Glasgow Coma Scale, systolic and diastolic blood pressures, prothrombin time-international normalized ratio (PT-INR), white blood cell count, hemoglobin, platelet count, serum creatinine, serum total bilirubin, and time from onset to baseline CT scan.

This study was approved by the following institutional review boards: Mie Chuo Medical Center institutional review board [permit number: MCERB-201926], Matsusaka Chuo General Hospital institutional review board [permit number: 232], Suzuka Kaisei Hospital institutional review board [permit number: 2020–05], and Mie University Hospital institutional review board [permit number: T2019-19]. Because this was a retrospective study, separate informed patient consent was waived by the following institutional review boards: Mie Chuo Medical Center institutional review board [permit number: MCERB-201926], Matsusaka Chuo General Hospital institutional review board [permit number: 232], Suzuka Kaisei Hospital institutional review board [permit number: 2020–05], and Mie University Hospital institutional review board [permit number: T2019-19]. All study protocols and procedures were conducted in accordance with the Declaration of Helsinki. This manuscript was prepared according to the standards for reporting of diagnostic accuracy (STARD) statement.

### Imaging analysis

CT scans were performed using 120 kVp with a thickness of 0.5–10.0 mm in the supine position. CT angiography was performed by injecting 50–100 ml of an iodinated contrast material at 3.5–5.0 ml/s; but not all patients underwent CT angiography. Manufacturers and models of CT scanners in the development cohort included Aquilion ONE (Canon Medical Systems, Ohtawara, Japan), Aquilion 64 (Canon Medical Systems), LightSpeed Plus (GE Medical Systems, Milwaukee, WI, USA), LightSpeed VCT (GE Medical Systems), BrightSpeed Elite (GE Medical Systems), and SOMATOM Definition Flash (SIEMENS Healthineers, Erlangen, Germany), and those in the validation cohort included Aquilion 64 and Discovery CT750 HD (GE Medical Systems).

The hemorrhage locations were categorized as basal ganglia, thalamus, lobe, brain stem, and cerebellum. The presence of intraventricular extension of hemorrhage was noted. The hematoma volume was calculated with the ABC/2 formula^27^. Hematoma expansion was defined as an increase in volume between baseline and follow-up CT scans exceeding 6 cm^3^ or 33% of the baseline volume^16–20,28^.

Intrahematoma hypodensities, irregular hematoma shape, and blend sign were identified as noncontrast CT markers. Intrahematoma hypodensities were defined as presence of any hypodense region encapsulated within the hematoma having any morphology and size, separated from the surrounding parenchyma^3,4,12,14^. Irregular hematoma shape was defined as presence of 2 or more hematoma edge irregularities^4,7,9,12^. Blend sign was defined as blending of relatively hypoattenuating area with adjacent hypoattenuating region within a hematoma with a well-defined margin and at least 18 Hounsfield units difference from these regions^4,6,8,12^. When available, CT angiography spot sign was evaluated, which was defined as follows: (1) ≥ 1 focus (attenuation ≥ 120 Hounsfield units) of any size and morphology of contrast pooling within a hematoma, and (2) discontinuous from normal or abnormal vasculature adjacent to the hematoma^15,29^. The CT markers were independently evaluated by 2 observers. When the evaluation by observers disagreed, the CT images were re-evaluated by both observers together, with consensus being developed.

### Inhospital management

After identification of ICH on baseline CT scan, continuous blood pressure monitoring and blood pressure-lowering treatment were initiated. Calcium channel blockers, mainly intravenous nicardipine, were administered as antihypertensive agents throughout the period between baseline and follow-up CT scans. The target systolic blood pressure was less than 140 mmHg or 180 mmHg.

### Statistical analysis

Continuous variables were summarized using a mean with standard deviation or a median with interquartile range and compared using Student’s t test or Mann–Whitney U test, depending on the distribution of the variable assessed by the Shapiro–Wilk test. Categorical variables were summarized using a count with percentages and compared using Fisher’s exact test.

To confirm the superiority of predictive models using ML over the previous scoring methods, the BAT, BRAIN, and 9-point scores in the validation cohort were calculated^16–19^. The receiver operating characteristic (ROC) curve was drawn, where the best cutoff value by the Youden’s index was determined. In each scoring method, accuracy, sensitivity, specificity, and the area under the ROC curve (AUC) for the prediction of hematoma expansion were computed. The AUC of the three scores and that of ML models were compared using DeLong test.

All statistical analyses were performed using EZR (Saitama Medical Center, Jichi Medical University, Saitama, Japan)^30^, which is a graphical user interface for R (The R Foundation for Statistical Computing, Vienna, Austria).

### Machine learning environment and algorithms

The programming language Python (version 3.7.8) and its libraries, NumPy (version 1.19.1), scikit-learn (version 0.23.2), XGBoost (version 1.2.0), imbalanced-learn (version 0.7.0), and matplotlib (version 3.3.1), were used for all data processing. The programming code was executed in Jupyter Notebook (version 6.0.3).

To develop predictive models, supervised ML algorithms were adopted, in which pairs of the input data and the output class were given to the algorithm, which found a way to generate the output class from the input data^31^. The k-nearest neighbors (k-NN) algorithm, logistic regression, support vector machines (SVMs), random forests, and XGBoost were selected as the supervised algorithms. The k-NN algorithm is the simplest ML algorithm, which finds k neighbors closest to a new observation in the stored training data and makes a prediction by assigning the majority class among these neighbors^31^. Logistic regression is a binary classifier, in which a linear model is included in a logistic function and the probability that a new observation is a member of each class is computed^31^. SVMs find the hyperplane that maximizes the margin between classes in the training data, making a prediction based on the distances to the support vectors and the importance of support vectors^31^. Random forests train many decision trees, where each tree only receives a bootstrapped observation of training data and each node only considers a subset of features when determining the best split, making a prediction in accordance with the averaged probabilities predicted by all the trees^31^. XGBoost is a gradient boosting algorithm, which works by building decision trees in a serial manner, where each tree tries to correct the mistakes of the previous one; and the probability is computed by summing the weight of the leaves to which a new observation belongs in each decision tree^31^. With each supervised algorithm, predictive model development using the patent data of the development cohort (training data set) and external validation using that of the validation cohort (test data set) were planned.

### Feature selection and scaling, and oversampling

Baseline clinical variables, CT findings including hemorrhage locations, intraventricular hematoma extension, baseline hematoma volume, and noncontrast CT markers, and target systolic blood pressure were applied as the input data, while hematoma expansion was applied as the output class.

Since there were 31 individual properties of the input data, which were called features, feature selection was performed to lead to simpler models that generalize better^31^. Firstly, univariate analyses with Student’s t test, Mann–Whitney U test, and Fisher’s exact test were performed between expansion and no expansion groups in the training data set. Secondly, the features were ranked in accordance with their P values. Finally, 5 to 10 features with the smallest P values were selected. Feature scaling was performed using standardization in SVMs, which required all the features to vary on a similar scale to perform well.

Given the imbalance of the output class distribution, random oversampling was employed. Random oversampling involved randomly selecting observations from the minority group with replacement and adding them to the training data set.

### Predictive model development and external validation

Each supervised ML algorithm was applied to the training data set with 5 to 10 selected features and all 31 features. In the predictive model development process, stratified 30-fold cross-validation was used to assess generalization performance, in which the training data set was split such that the proportions between output classes were the same in each fold as they were in the whole training data set^31^. The hyperparameters were tuned manually in each algorithm as shown in Table 1 to improve generalization performance, while the other hyperparameters not listed in Table 1 were used as default.

**Table 1:** Manually tuned hyperparameters and their values in each machine learning algorithm.

	Hyperparameter	Value
k-nearest neighbors algorithm	n_neighbors	3, 5, 7, 9, 11, 13
Logistic regression	C	0.001, 0.01, 0.1, 1, 10, 100
Support vector machines	C	0.001, 0.01, 0.1, 1, 10, 100
	Gamma	0.001, 0.01, 0.1, 1, 10, 100
Random forests	n_estimators	75, 125, 175, 225
	max_depth	2, 3, 4, 5, 6, 7
XGBoost	num_round	10, 20, 30
	eta	0.01, 0.1
	max_depth	3, 4, 5, 6, 7
	min_child_weight	1, 2, 3, 4, 5
	Colsample_bytree	0.7, 0.8, 0.9
	Subsample	0.7, 0.8, 0.9
	Gamma	0, 0.1, 0.2
	Alpha	0, 0.01, 0.1

After the model development, each model was evaluated for its performance on the test data set as external validation, where accuracy, sensitivity, specificity, and the AUC for the prediction of hematoma expansion were computed.

## Results

**Figure 1:**
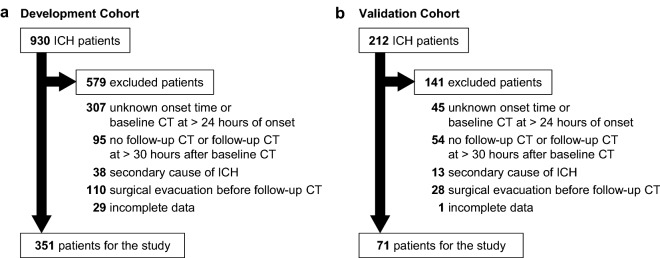
A flow chart indicating the included and excluded patients in the development cohort (**a**) and the validation cohort (**b**). ICH indicates intracerebral hemorrhage.

After application of the inclusion and exclusion criteria, 351 of 930 patients in the development cohort and 71 of 212 patients in the validation cohort were evaluated (Fig. 1). Hematoma expansion occurred in 71 patients (20.2%) in the development cohort and in 26 patients (36.6%) in the validation cohort (Table 2).

**Table 2:** Characteristics of the development and validation cohorts.

	Development cohort (n = 351)	Validation cohort (n = 71)	P Value
Age, y	73 (63–83)	70 (56–78)	0.017*
Sex (male)	215 (61.3)	42 (59.2)	0.790^†^
History of intracerebral hemorrhage	21 (6.0)	3 (4.2)	0.780^†^
History of cerebral infarction	53 (15.1)	3 (4.2)	0.012^†^
History of ischemic heart disease	16 (4.6)	3 (4.2)	1.000^†^
History of hypertension	207 (59.0)	43 (60.6)	0.895^†^
History of diabetes mellitus	77 (21.9)	12 (16.9)	0.426^†^
History of dyslipidemia	133 (37.9)	14 (19.7)	0.004^†^
Anticoagulant use	26 (7.4)	11 (15.5)	0.037^†^
Antiplatelet use	72 (20.5)	10 (14.1)	0.251^†^
Glasgow Coma Scale	15 (12–15)	14 (11–15)	0.373*
Systolic blood pressure, mmHg	182.5 ± 32.7	191.1 ± 32.6	0.042^‡^
Diastolic blood pressure, mmHg	100.4 ± 22.3	104.1 ± 22.1	0.205^‡^
PT-INR	1.00 (0.95–1.05)	0.94 (0.91–1.04)	0.002*
White blood cell count, 10^6^/mL	7.50 (5.80–9.94)	8.11 (5.76–10.33)	0.625*
Hemoglobin, mg/dL	13.6 ± 2.0	13.9 ± 2.1	0.243^‡^
Platelet count, 10^6^/mL	211.4 ± 62.3	217.9 ± 55.7	0.415^‡^
Serum creatinine, mg/dL	0.73 (0.60–0.90)	0.70 (0.55–0.86)	0.177*
Serum total bilirubin, mg/dL	0.7 (0.5–0.9)	0.7 (0.6–0.8)	0.702*
Time from onset to baseline CT scan, h	2 (1–4)	1 (1–2)	< 0.001*
**Hemorrhage locations**			
Basal ganglia	120 (34.2)	31 (43.7)	0.137^†^
Thalamus	115 (32.8)	23 (32.4)	1.000^†^
Lobe	73 (20.8)	7 (9.9)	0.031^†^
Brain stem	15 (4.3)	6 (8.5)	0.141^†^
Cerebellum	28 (8.0)	4 (5.6)	0.628^†^
Intraventricular hematoma extension	144 (41.0)	30 (42.3)	0.895^†^
Baseline hematoma volume, mL	11.9 (4.9–29.1)	16.8 (6.2–27.9)	0.190*
Intrahematoma hypodensities	123 (35.0)	37 (52.1)	0.010^†^
Irregular hematoma shape	211 (60.1)	50 (70.4)	0.110^†^
Blend sign CT angiography spot sign**	29 (8.3) 6 (7.1)	5 (7.0) 13 (38.2)	1.000^†^ < 0.001^†^
Target systolic blood pressure			< 0.001^†^
Less than 140 mmHg	183 (52.1)	60 (84.5)	
Less than 180 mmHg	168 (47.9)	11 (15.5)	
Hematoma expansion	71 (20.2)	26 (36.6)	0.005^†^

Data are presented as n (%), mean ± standard deviation, or median (interquartile range).

CT = computed tomography; PT-INR = prothrombin time-international normalized ratio.

*Mann–Whitney U test between the development and validation cohorts.

^†^Fisher’s exact test between the development and validation cohorts.

^‡^Student’s t test between the development and validation cohorts.

**CT angiography is not performed in all patients.

On comparison between expansion and no expansion groups in the development cohort, 10 variables with the smallest P values were baseline hematoma volume, intrahematoma hypodensities, PT-INR, anticoagulant use, lobar hemorrhage, irregular hematoma shape, platelet count, sex, time from onset to baseline CT scan, and cerebellar hemorrhage in increasing order (Table 3): these were used as selected features.

**Table 3:** Univariate analyses between expansion and no expansion groups in the development cohort.

	Expansion (n = 71)	No Expansion (n = 280)	P Value
Age, y	76 (67–84)	71 (62–82)	0.086*
Sex (male)	54 (76.1)	161 (57.5)	0.004^†^
History of intracerebral hemorrhage	8 (11.3)	13 (4.6)	0.048^†^
History of cerebral infarction	10 (14.1)	43 (15.4)	0.855^†^
History of ischemic heart disease	7 (9.9)	9 (3.2)	0.025^†^
History of hypertension	37 (52.1)	170 (60.7)	0.224^†^
History of diabetes mellitus	13 (18.3)	64 (22.9)	0.521^†^
History of dyslipidemia	20 (28.2)	113 (40.4)	0.075^†^
Anticoagulant use	13 (18.3)	13 (4.6)	< 0.001^†^
Antiplatelet use	20 (28.2)	52 (18.6)	0.099^†^
Glasgow Coma Scale	14 (11–15)	15 (12–15)	0.108*
Systolic blood pressure, mmHg	176.9 ± 29.3	183.9 ± 33.4	0.110^‡^
Diastolic blood pressure, mmHg	96.3 ± 22.1	101.4 ± 22.3	0.085^‡^
PT-INR	1.02 (0.98–1.11)	0.99 (0.94–1.04)	< 0.001*
White blood cell count, 10^6^/mL	6.80 (5.57–8.41)	7.77 (5.94–10.10)	0.033*
Hemoglobin, mg/dL	13.1 ± 1.8	13.7 ± 2.0	0.031^‡^
Platelet count, 10^6^/mL	190.8 ± 58.1	216.7 ± 62.4	0.002^‡^
Serum creatinine, mg/dL	0.73 (0.63–0.91)	0.73 (0.59–0.90)	0.634*
Serum total bilirubin, mg/dL	0.7 (0.5–1.0)	0.7 (0.5–0.9)	0.405*
Time from onset to baseline CT scan, h	1 (1–3)	2 (1–4)	0.006*
**Hemorrhage locations**			
Basal ganglia	27 (38.0)	93 (33.2)	0.484^†^
Thalamus	16 (22.5)	99 (35.4)	0.047^†^
Lobe	26 (36.6)	47 (16.8)	< 0.001^†^
Brain stem	1 (1.4)	14 (5.0)	0.322^†^
Cerebellum	1 (1.4)	27 (9.6)	0.024^†^
Intraventricular hematoma extension	26 (36.6)	118 (42.1)	0.421^†^
Baseline hematoma volume, mL	30.7 (17.1–57.2)	9.5 (4.0–19.7)	< 0.001*
Intrahematoma hypodensities	43 (60.6)	80 (28.6)	< 0.001^†^
Irregular hematoma shape	55 (77.5)	156 (55.7)	0.001^†^
Blend sign	9 (12.7)	20 (7.1)	0.148^†^
Target systolic blood pressure			0.894^†^
Less than 140 mmHg	38 (53.5)	145 (51.8)	
Less than 180 mmHg	33 (46.5)	135 (48.2)	

Data are presented as n (%), mean ± standard deviation, or median (interquartile range).

CT = computed tomography; PT-INR = prothrombin time-international normalized ratio.

*Mann–Whitney U test between expansion and no expansion groups.

^†^Fisher’s exact test between expansion and no expansion groups.

^‡^Student’s t test between expansion and no expansion groups.

The k-NN algorithm achieved the highest AUC of 0.790 (95% confidence interval [CI], 0.693–0.886) among all ML models, where 9 selected features were used and the hyperparameter n_neighbors was 5 (Table 4). Logistic regression yielded the AUC of 0.674 (95% CI, 0.563–0.784) when 6 selected features were used, and C was 0.1. SVMs yielded the AUC of 0.740 (95% CI, 0.634–0.846) when all 31 features were used, and C and gamma were 1 and 0.01, respectively. Random forests yielded the AUC of 0.741 (95% CI, 0.633–0.849) when all 31 features were used, and n_estimators and max_depth were 125 and 3, respectively. XGBoost yielded the AUC of 0.732 (95% CI, 0.623–0.841) when 9 selected features were used, and num_round, eta, max_depth, min_child_weight, colsample_bytree, subsample, gamma, and alpha were 20, 0.1, 4, 4, 0.9, 0.8, 0.1, and 0, respectively.

**Table 4:** Test characteristics of previously reported scoring methods and machine learning models in the validation cohort.

	Accuracy	Sensitivity	Specificity	AUC
**Previous scoring methods**				
BAT score	0.606 (0.483–0.720)	0.654 (0.443–0.828)	0.578 (0.422–0.723)	0.616 (0.497–0.734)
BRAIN score	0.620 (0.497–0.732)	0.885 (0.698–0.976)	0.467 (0.317–0.621)	0.676 (0.579–0.772)
9-point score	0.690 (0.569–0.795)	0.538 (0.334–0.734)	0.778 (0.629–0.888)	0.658 (0.543–0.774)
**Machine learning models**				
k-nearest neighbors algorithm	0.775 (0.660–0.865)	0.846 (0.651–0.956)	0.733 (0.581–0.854)	0.790 (0.693–0.886)
Logistic regression	0.648 (0.525–0.758)	0.769 (0.564–0.910)	0.578 (0.422–0.723)	0.674 (0.563–0.784)
Support vector machines	0.732 (0.614–0.831)	0.769 (0.564–0.910)	0.711 (0.557–0.836)	0.740 (0.634–0.846)
Random forests	0.775 (0.660–0.865)	0.615 (0.406–0.798)	0.867 (0.732–0.949)	0.741 (0.633–0.849)
XGBoost	0.732 (0.614–0.831)	0.731 (0.522–0.884)	0.733 (0.581–0.854)	0.732 (0.623–0.841)

Data are presented as value (95% confidence interval).

AUC = area under the receiver operating characteristic curve.

The best cutoff values in the previous scoring methods were 3 in the BAT score, 9 in the BRAIN score, and 4 in the 9-point score. Although the BRAIN score achieved the highest AUC of 0.676 (95% CI, 0.579–0.772) among all previous scoring methods, the k-NN algorithm that achieved the best performance of all ML models showed higher AUC than the BRAIN score (0.790 vs. 0.676; p = 0.016) (Table 4).

## Discussion

We developed and validated ML predictive models of hematoma expansion in acute ICH. The models demonstrated good predictive ability, showing better performance than the previous scoring methods. Multicenter data and multivendor CT images were used for model development, so that the models were generalizable and widely applicable.

Thirty-one features, consisting of baseline clinical variables, CT findings, and target systolic blood pressure, were put into the model development process. Clinical variables only contained general patient information and blood test findings. Thus, they could be easily collected in clinical practice. All CT findings were obtained from noncontrast CT scans; and CT scan data included those performed with a thickness of 0.5–10.0 mm. Although the spot sign, which is also included in the 9-point score, is useful for predicting hematoma expansion, CT angiography is available in a limited number of hospitals. Additionally, although noncontrast CT markers are usually evaluated with a thickness of 5.0 mm, in clinics or developing countries, CT scans are not uncommonly performed with a thickness of more than 5 mm. Therefore, in order that predictive models could be used in many hospitals and countries, we acquired and analyzed CT scan data for such conditions. We experimentally included target systolic blood pressure in the features because it could be determined at admission. However, there was no statistical difference regarding target systolic blood pressure between expansion and no expansion groups in the development cohort. Therefore, target systolic blood pressure was not included in the features of the best ML model.

Feature selection was performed to develop simpler ML predictive models. When developing models using many features, or a high-dimensional data set, models become complex and the chance of overfitting increases^31^. There are three basic strategies for selecting features: model-based selection, iterative selection, and univariate analysis^31^. Model-based selection utilizes supervised ML models such as linear models and decision tree-based models to judge the importance of each feature. In iterative selection, a series of models for feature selection are built, where the features with higher importance are selected. These methods consider all features at once and may be able to capture interactions between features. However, when the performance of the models for feature selection is low, selected features could be unreliable. Univariate analysis was the one that we chose in this study, where a correlation between individual features was ignored and therefore features that were only informative when combined with other features were discarded. Still, we showed good performance in the best ML model using univariate analysis, but there may be better feature selection methods. However, there is one caveat: elaborate feature selection may lead to overfitting, resulting in reducing model performance.

We have made the raw data and the programming code of ML algorithms available on the websites to ensure reproducibility of the developed models: we believe that this is the most important point for the clinical studies using ML. There may be better ML approaches than what we have shown in this study, and better ML algorithms that can achieve higher performance may be created in the future. By using ML approaches, we can easily add the data of other facilities and develop more robust and reliable ML models. With the maturity of ML technology and its usage environment, it is becoming easier for clinicians to learn ML and apply it to clinical research. We hope that our data and algorithms will be widely used and applied to new analyses.

ML approaches have been used in medical research and often perform better than classical statistical models^21,22^. In this study, even though there were some statistical differences in patient characteristics between the development and validation cohorts (Table 2), the developed ML models showed better predictive ability than the previous scoring methods, such as the BAT, BRAIN, and 9-point scores, in the validation cohort^16–19^.

Several clinical studies have investigated the relationships between lowering of blood pressure and the outcome in patients or hematoma expansion though no conclusion has been reached yet^32–35^. However, the ultra-early lowering of blood pressure may benefit patients with acute ICH^36^. Moreover, anticoagulant reversal may reduce hematoma expansion^37^. The developed ML models in this study may be useful, especially in ultra-early phase or when anticoagulants are given, for selecting patients who require more careful treatment.

A few limitations should be noted. First, more patients in the development and validation cohorts are needed to achieve more robust quality and more satisfactory performance of ML predictive models. It is hard to determine the appropriate number of patients in ML analyses because it depends on the quality of the input data. However, efforts are required to increase the number of patients and to make sure that model performance have reached a plateau irrespective of an increase of the number of patients^38^. Second, CT findings were evaluated by humans. If we utilize an artificial neural network for analyzing CT scan data, we can create hybrid models that unify analyses of imaging data and clinical variables within a ML pipeline. The hybrid models are likely to achieve higher predictive performance. However, as a serious problem, brain image data usually contain face information, which cannot easily be shared.

In conclusion, we developed widely applicable predictive models of hematoma expansion in acute ICH by applying ML algorithms to clinical data and noncontrast CT findings. The models showed better performance than the previous scoring methods. We have made the raw data and the programming code available on the websites so that anyone can utilize and improve the models.

## Data Availability

The raw clinical data of the development and validation cohorts in comma-separated values format are deposited in OSF (https://osf.io/etw9j/download). The programming code of ML algorithms is available on GitHub (https://github.com/taniokas-neuro/predict-hematoma-expansion). The data that supports the findings of this study are available from the corresponding author.
